# Determining Optimal Feature-Combination for LDA Classification of Functional Near-Infrared Spectroscopy Signals in Brain-Computer Interface Application

**DOI:** 10.3389/fnhum.2016.00237

**Published:** 2016-05-25

**Authors:** Noman Naseer, Farzan M. Noori, Nauman K. Qureshi, Keum-Shik Hong

**Affiliations:** ^1^Department of Mechatronics Engineering, Air UniversityIslamabad, Pakistan; ^2^Department of Cogno-Mechatronics, School of Mechanical Engineering, Pusan National UniversityBusan, Korea

**Keywords:** functional near-infrared spectroscopy, brain-computer interface, optimal feature selection, linear discriminant analysis, binary classification, mental arithmetic

## Abstract

In this study, we determine the optimal feature-combination for classification of functional near-infrared spectroscopy (fNIRS) signals with the best accuracies for development of a two-class brain-computer interface (BCI). Using a multi-channel continuous-wave imaging system, mental arithmetic signals are acquired from the prefrontal cortex of seven healthy subjects. After removing physiological noises, six oxygenated and deoxygenated hemoglobin (HbO and HbR) features—mean, slope, variance, peak, skewness and kurtosis—are calculated. All possible 2- and 3-feature combinations of the calculated features are then used to classify mental arithmetic vs. rest using linear discriminant analysis (LDA). It is found that the combinations containing mean and peak values yielded significantly higher (*p* < 0.05) classification accuracies for both HbO and HbR than did all of the other combinations, across all of the subjects. These results demonstrate the feasibility of achieving high classification accuracies using mean and peak values of HbO and HbR as features for classification of mental arithmetic vs. rest for a two-class BCI.

## Introduction

Engineering principles and techniques are nowadays becoming crucial aspects of development in the medical fields. Key examples are the diagnosis and cure of various diseases in the human body. Over the past few decades, the brain-computer interface (BCI), as utilized with computers and other external devices, has become an indispensable medium of communication for patients suffering from amyotrophic lateral sclerosis (ALS), locked-in syndrome (LIS) and other physical disabilities. Brain-signal acquisition methods for BCI are either invasive or non-invasive. Invasive brain-signal acquisition methods, albeit allowing for acquisition of fine-quality brain signals, incur the risks of surgery (Wester et al., 2009; Thongpang et al., 2011; Viventi et al., 2011). Non-invasive methods, therefore, are preferred. There are several types of non-invasive brain-signal acquisition methods, including electroencephalography (EEG) (Wolpaw et al., 2002; Salvaris and Sepulveda, 2010; Choi, 2013), functional magnetic resonance imaging (fMRI) (Enzinger et al., 2008; Sorger et al., 2009) and functional near infrared spectroscopy (fNIRS) (Ferrari et al., 1985; Kato et al., 1993; Coyle et al., 2004, 2007; Naito et al., 2007; Naseer and Hong, 2013; Naseer et al., 2014). fNIRS has better spatial resolution than most of the EEG systems (Hu et al., 2012; Hong et al., 2014). Furthermore, fNIRS signals are free of electrical noises and use of conductive gels. fMRI provides a good spatial resolution, though the equipment is bulky and, therefore, not feasible for a portable BCI. For recent BCI applications rather, fNIRS has been utilized owing to its balanced spatial and temporal resolutions, low noise, safety and overall ease of use. Indeed, it has been shown to work well for binary communication with high classification accuracies (Naseer et al., 2014). fNIRS has become a neuroimaging technique which is contributing in making advances toward the understanding of the human brain functionality (Irani et al., 2007; Ferrari and Quaresima, 2012; Hong and Nguyen, 2014; Hong and Naseer, 2016; Hong and Santosa, 2016).

fNIRS consists of near-infrared (NI) emitters that emit light within the 650~1000 nm wavelength range in order to measure changes in the concentrations of oxygenated hemoglobin and deoxygenated hemoglobin (△*c*_HbO_(*t*) and △*c*_HbR_(*t*)) (Villringer et al., 1993; Hoshi et al., 1994; Hoshi and Tamura, 1997). Oxygenated and deoxygenated hemoglobin (HbO and HbR) have diverse absorption spectra in the NI range; therefore, the association between the exiting- and incident-photon intensities can be used to calculate △*c*_HbO_(*t*) and △*c*_HbR_(*t*) on the photon paths by application of the modified Beer-Lamberts law (Delpy et al., 1988; Sassaroli and Fantini, 2004). Since Jobsis (1977) introduced the principle of near-infrared spectroscopy, it has been effectively employed for functional and structural brain imaging as well as BCI purposes (Hu et al., 2010, 2011, 2012, 2013; Cutini et al., 2012; Aqil et al., 2012a,b; Bhutta et al., 2014, 2015; Hong et al., 2014; Khan et al., 2014).

With fNIRS-BCI systems, the user elicits distinct brain-signal patterns by performing different mental tasks such as motor imagery (Coyle et al., 2004, 2007), mental arithmetic (MA) (Naito et al., 2007; Bauernfeind et al., 2008, 2011; Utsugi et al., 2008), music imagery (Naito et al., 2007; Power et al., 2010; Falk et al., 2011) and others. Pattern recognition techniques are then used to identify and recognize these signals. The related command signals can then be generated to communicate with a computer or external device in ways intended by the user. After the suitable signals are acquired from a specific brain region, noise removal techniques are used to remove the noises such as experimental, instrumental and physiological (cardiac and respiratory activities) (Kirilina et al., 2012; Santosa et al., 2013; Bajaj et al., 2014). Since these noises are uncorrelated with the experimental paradigm, the effect of these noises in fNIRS signals might yield to false or biased conclusions (Cui et al., 2010b; Santosa et al., 2014; Naseer and Hong, 2015a). The next step is feature extraction, based on which the signals are classified. In the relevant previous studies, the different statistical properties of time-domain signals have been used as features for classification; those properties include the mean, variance, slope, kurtosis, peak value and skewness, among others. To date, however, optimal feature-combination selection for the best classification accuracies has not been demonstrated.

The objective of the present study was to determine the optimal 2- and 3-feature combinations (among mean, variance, slope, kurtosis, peak value, and skewness) that yield the best “mental arithmetic task vs. rest” classification accuracies for a two-class fNIRS-BCI using linear discriminant analysis (LDA). After acquiring fNIRS signals representing mental arithmetic tasks or rest from the prefrontal cortex, noises were removed using a notch filter. Then, the six features noted above were calculated according to the △*c*_HbO_(*t*) and △*c*_HbR_(*t*) signals; all of the possible 2- and 3-feature combinations of those features were used to train the LDA (Lotte et al., 2007; Luu and Chau, 2009; Moghimi et al., 2012; Hong et al., 2014) classifier. For each of those combinations, the classification performance was evaluated using 10-fold cross-validation.

## Materials and methods

### fNIRS data acquisition

A multichannel continuous-wave imaging system (DYNOT: DYnamic Near-infrared Optical Tomography; two wavelengths: 760 and 830 nm; NIRx Medical Technologies, NY) was used to acquire brain signals at a sampling rate of 1.81 Hz. The continuous-wave fNIRS detects the △*c*_HbO_(*t*) and △*c*_HbR_(*t*) in the microvessels of the cortex according to the modified Beer-Lamberts law:

(1) $$\begin{array}{l} \left[\begin{array}{c} {△c}_{\text{HbO}}\left(t\right)\\ {△c}_{\text{HbR}}\left(t\right) \end{array}\right]=\frac{1}{l\times d}{\left[\begin{array}{c} \alpha_{\text{HbO}}\left(\lambda_{1}\right)\\ \alpha_{\text{HbO}}\left(\lambda_{2}\right) \end{array}\begin{array}{c} \alpha_{\text{HbR}}\left(\lambda_{1}\right)\\ \alpha_{\text{HbR}}\left(\lambda_{2}\right) \end{array}\right]}^{-1}\left[\begin{array}{c} △A\left(t,\lambda_{1}\right)\\ △A\left(t,\lambda_{2}\right) \end{array}\right] \end{array}$$

where △*A*(*t*; ;λ_*j*_) (*j* = 1.2) is the unit-less absorbance (optical density) variation of a light emitter of wavelength λ_*j*_, *a*_HbX_(λ_*j*_) is the extinction coefficient of HbX (HbO and HbR) in μM^−1^ mm^−1^, *d* is the unit-less differential path length factor (DPF), and *l* is the distance (in millimeters) between emitter and detector. The placement of the fNIRS optodes plays a crucial role in signal measurement, since a longer emitter-detector distance makes for greater imaging depth (McCormick et al., 1992). Usually an emitter-detector distance of around 2.5~3.5 cm is applied, because a distance less than 2 cm might result in only superficial-layer signal capture, while a distance more than 4 cm usually is too weak to be usable (Gratton et al., 2006).

**Figure 1:**
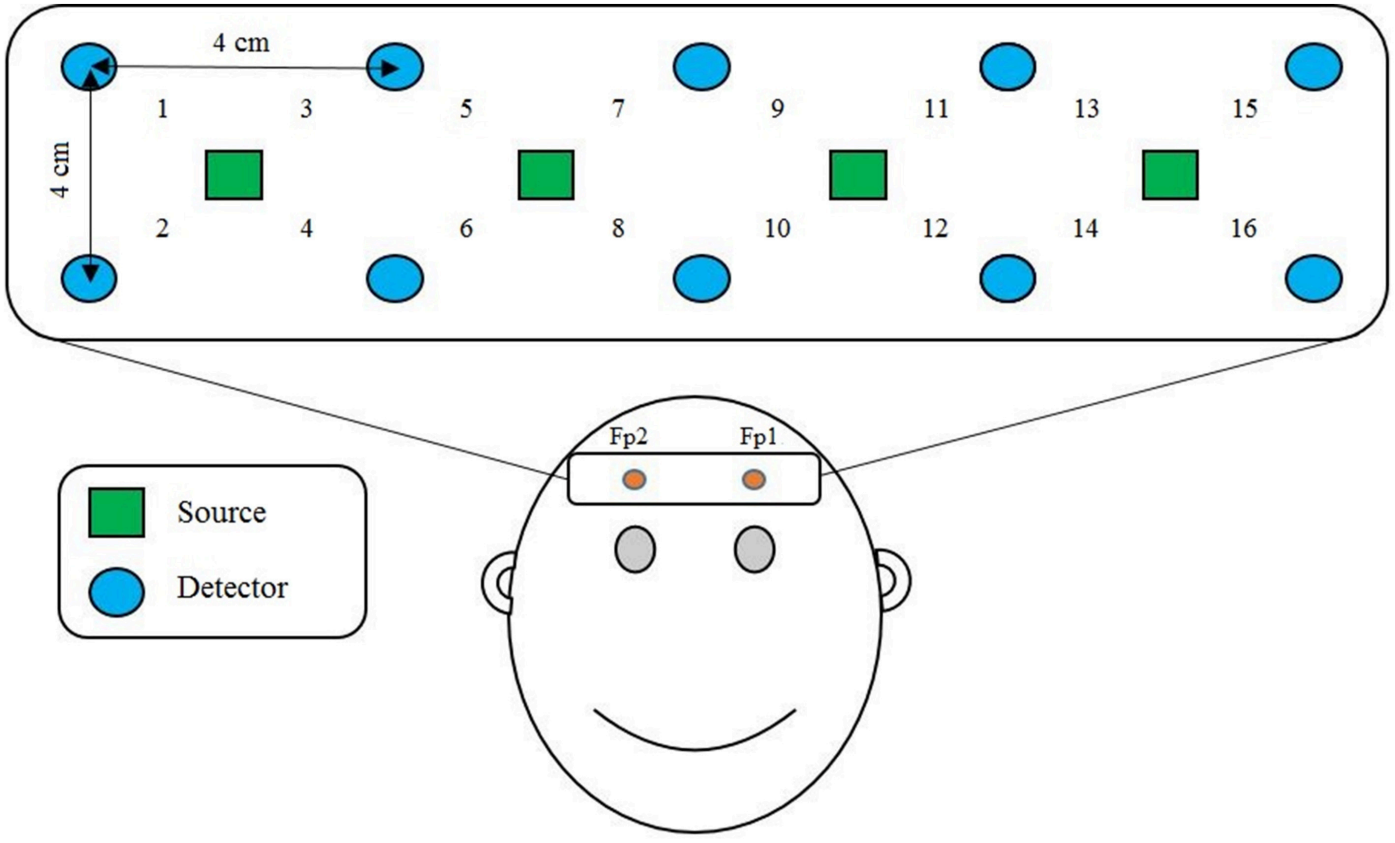
**Optode placement and channel location on the prefrontal cortex**. Fp1 and Fp2 are the reference points of the International 10–20 system.

We applied a 2.8 cm emitter-detector distance, as shown in Figure 1, in order to acquire brain signals resulting from mental arithmetic tasks. To that end, 4 near-infrared light emitters and 10 detectors were positioned over the prefrontal cortex, as it is known that mental arithmetic activates the prefrontal cortex of the brain (Ayaz et al., 2013, 2014; Khan et al., 2014; Di Domenico et al., 2015).

### Experimental procedure

Seven male subjects (mean age 30.5 ± 5 years) participated in the experiments. The subjects were seated in a comfortable chair in front of a computer monitor. They were asked to relax and restrict their head movements as much as possible while performing a mental arithmetic task. The first 44 s was a rest period to set up the baseline condition; this was followed by a 44 s mental arithmetic task period, which was followed in turn by another 44 s rest period to allow the signals to return to the baseline values before the start of the next trial. The above sequence was repeated 5 times for a total experimental duration of 440 s for each subject. During the mental arithmetic task period, the participants performed a series of mental arithmetic calculations that appeared on the monitor in a pseudo-random order. These calculations consisted of subtraction of a two-digit number (between 10 and 20) from a three-digit number throughout the task period with successive subtraction of a two-digit number from the result of the previous subtraction (e.g., 244−14, 240−11, 229−16, etc.)(Power et al., 2011; Naseer et al., 2014; Naseer and Hong, 2015b). During the rest period the subjects were asked to relax and continue looking at the monitor. The experiments were conducted in accordance with the latest declaration of Helsinki and a verbal consent was taken from all the subjects after explaining the experimental paradigm. The work was approved by the Institutional Review Board of Pusan National University.

### Signal processing and classification

The optical-density signals acquired were first converted to △*c*_*HbX*_(*t*) signals using Equation (1). Then, they were filtered using a notch filter with band-reject ranges of 1~1.2 Hz, 0.3~0.4 Hz, and below 0.1 Hz (Naseer and Hong, 2015a) in order to reduce physiological noises due to heartbeat, respiration, and Mayer waves and low frequency fluctuations (Bajaj et al., 2013), respectively. The frequency range considered for analysis was 0.1~0.3 Hz. Detrending of the data was performed using NIRS-SPM (Ye et al., 2009).

In this study, only the △*c*_HbO_(*t*) and △*c*_HbR_(*t*) signals were considered as features (Rejer, 2015) for classification. Although, fNIRS also provides changes in total concentration, △*c*_HbT_(*t*). However, since △*c*_HbT_(*t*) is just the addition of △*c*_HbO_(*t*) and △*c*_HbR_(*t*), it does not provide extra discriminative information and, therefore, has not been used as a feature for classification in most of the previous fNIRS-BCI studies (Bhutta et al., 2014; Khan et al., 2014; Santosa et al., 2014; Naseer and Hong, 2015b).

As classification features, all of the possible 2- and 3-feature combinations of signal slope, signal mean, signal variance, signal peak, signal kurtosis and signal skewness were considered as in Khan and Hong (2015). The signal mean of △HbO and △HbR are calculated as:

$$\begin{array}{l} M=\frac{1}{N}\overset{N}{\underset{i=1}{\sum }}X_{i} \end{array}$$

where *M* is the mean value, *N* is the number of observations and *X*_*i*_ represents the HbO or HbR data. The variance is calculated as follows:

$$\begin{array}{l} var\left(X\right)=\frac{\sum {\left(X-\mu \right)}^{2}}{N} \end{array}$$

where *var* is the variance, μ is the mean value of *X*. The skewness is computed as follows:

$$\begin{array}{l} skew\left(X\right)=E\left[{\left(\frac{X-\mu }{\sigma }\right)}^{3}\right] \end{array}$$

where *skew* is the skewness,*E* is the expected value of *X* and σ is the standard deviation of *X*. The kurtosis is computed as follows:

$$\begin{array}{l} kurt\left(X\right)=E\left[{\left(\frac{X-\mu }{\sigma }\right)}^{4}\right]. \end{array}$$

The signal peak is estimated using the Matlab *max* function. The signal slope is determined by fitting a line to all the data points during the mental arithmetic and rest using *polyfit* function in Matlab. These features were calculated for the mental arithmetic and rest periods across all 16 channels. The aim of classification was to decode the subject's state as “mental arithmetic task” or “rest task” with maximal accuracy. All of the feature values were scaled between 0 and 1 using the equation:

$$\begin{array}{l} x\prime =\frac{x-\text{min}\left(x\right)}{\text{max}\left(x\right)-\text{min}\left(x\right)} \end{array}$$

where *x*∈*R*^*n*^ denotes the original feature vales, *x*′ denotes the rescaled feature values between 0 and 1, max (*x*) is the largest value, and min (*x*) is the smallest value.

**Figure 2:**
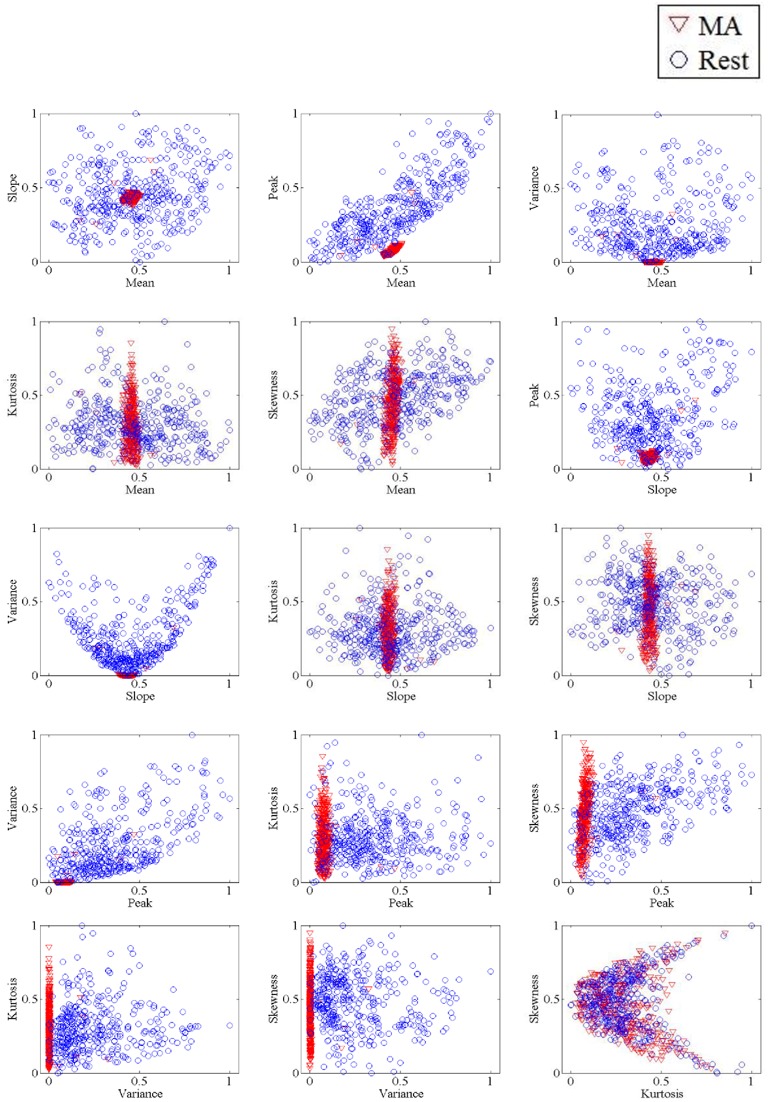
**The 2-dimensional feature spaces of Subject 1 for all combinations of HbO features**.

The method utilized in the present study to classify all possible 2- and 3-feature combinations of features extracted from the △*c*_HbO_(*t*) and △*c*_*HbR*_(*t*)signals was classified using LDA. LDA, a linear classifier, uses hyper-planes to separate the diverse classes of data (Lotte et al., 2007). The dividing hyper-plane is intended to maximize the separation between the class mean and minimize the inter-class variance. Owing to its ease of use and execution speed, LDA performs well in various BCI problems (Lotte et al., 2007; Salvaris and Sepulveda, 2010; Power et al., 2012a,b). Each classification performance was evaluated by 10-fold cross-validation over the course of 10 runs. Figure 2 shows the 2-dimensional feature spaces of subject 1 for all combinations.

## Results

For each subject, the classification accuracies obtained for all possible combinations of 2- and 3-features extracted using △*c*_HbO_(*t*) and △*c*_HbR_(*t*) are shown in Tables 1–4, respectively. The classification accuracies acquired using the 2-feature combination of peak and mean values were 93.0 and 89.9% using △*c*_HbO_(*t*) and △*c*_HbR_(*t*) signals, respectively. The classification accuracies obtained using the 3-feature combinations were correspondingly higher in the combinations including mean and peak values. In fact, these accuracies were higher as compared with all other possible combinations across all subjects for both △*c*_HbO_(*t*) and △*c*_HbR_(*t*) signals. To verify that the higher classification accuracies acquired using peak and mean values were statistically significant, we applied the permutation test. The *p*-values obtained using mean and peak values vs. all other combinations were less than 0.05 for both △*c*_HbO_(*t*) and △*c*_HbR_(*t*) signals, which established that the performance of the mean and peak value combination was statistically significant.

**Table 1:** **The classification accuracies of all 2-feature combinations obtained from HbO signals for all subjects**.

**Feature combination**	**S1**	**S2**	**S3**	**S4**	**S5**	**S6**	**S7**
Mean and Slope	53.82	50.81	50.69	59.22	59.84	55.21	59.84
Mean and Peak	94.61	96.48	90.71	91.96	90.96	91.96	94.85
Mean and Variance	86.57	87.21	81.93	82.93	82.81	75.53	83.43
Slope and Peak	87.07	83.31	80.92	85.44	83.81	83.56	81.18
Slope and Variance	86.95	88.71	83.43	82.81	81.81	76.78	80.55
Peak and Variance	89.71	89.96	83.56	87.71	87.21	83.68	81.31
Peak and Skewness	89.08	83.44	80.55	86.71	81.81	83.06	81.05
Mean and Skewness	48.11	49.56	49.81	53.07	52.94	51.94	50.31
Slope and Skewness	47.43	50.31	47.81	53.58	52.57	54.21	50.06
Kurtosis and Skewness	46.17	48.55	51.56	54.21	48.93	53.58	50.56
Variance and Skewness	87.82	88.58	82.31	83.18	81.55	78.29	84.19
Peak and Kurtosis	86.82	82.43	80.93	85.57	83.93	82.06	81.05
Mean and Kurtosis	46.92	46.67	51.44	53.71	49.05	52.07	48.43
Slope and Kurtosis	47.55	45.29	53.45	54.07	52.19	49.18	48.18
Variance and Kurtosis	87.45	88.33	82.18	83.31	82.31	82.18	85.95

**Table 2:** **The classification accuracies of all 2-feature combinations obtained from HbR signals for all subjects**.

**Feature combination**	**S1**	**S2**	**S3**	**S4**	**S5**	**S6**	**S7**
Mean and Slope	56.83	54.45	61.61	59.59	55.33	56.71	62.86
Mean and Peak	92.34	92.59	90.84	91.84	88.71	86.07	87.07
Mean and Variance	82.43	86.82	82.93	85.94	82.55	79.92	79.54
Slope and Peak	86.07	86.32	79.67	85.44	85.19	83.06	84.69
Slope and Variance	79.79	87.32	82.31	85.82	80.55	76.41	77.91
Peak and Variance	85.44	86.07	82.93	87.21	86.32	76.91	84.31
Peak and Skewness	88.33	87.21	84.44	85.94	84.94	80.81	85.44
Mean and Skewness	51.69	51.31	53.32	52.82	47.05	54.71	59.47
Slope and Skewness	52.44	52.07	57.34	51.31	51.94	47.55	54.83
Kurtosis and Skewness	52.94	48.81	55.21	49.43	45.42	56.46	52.69
Variance and Skewness	82.55	81.93	83.81	86.71	80.92	77.03	78.16
Peak and Kurtosis	86.95	83.81	81.43	86.07	85.69	78.67	86.44
Mean and Kurtosis	50.06	54.57	55.33	45.42	48.55	60.47	57.59
Slope and Kurtosis	51.81	49.43	54.21	48.55	49.31	56.21	59.09
Variance and Kurtosis	86.32	85.94	85.44	86.71	82.05	78.16	78.41

**Table 3:** **The classification accuracies of all 3-feature combinations obtained from HbO signals for all subjects**.

**Feature combination**	**S1**	**S2**	**S3**	**S4**	**S5**	**S6**	**S7**
Mean, Peak, and Slope	94.47	96.48	90.46	91.96	90.58	92.34	94.35
Mean, Peak, and Kurtosis	95.15	96.36	90.96	92.09	91.96	93.22	94.98
Mean, Peak, and Skewness	94.98	96.61	90.58	93.09	92.34	92.47	94.85
Mean, Peak, and Variance	94.35	96.98	91.21	92.34	91.84	91.84	94.73
Peak, Slope, and Skewness	89.08	84.19	79.79	86.95	85.44	83.93	81.43
Peak, Kurtosis, and Variance	89.83	89.58	84.06	87.07	87.45	84.06	81.92
Peak, Slope, and Variance	90.08	90.21	84.31	87.72	86.71	83.68	80.92
Variance, Slope, and Kurtosis	87.57	89.08	82.68	82.43	82.68	78.92	81.55
Variance, Slope, and Mean	87.82	87.95	83.06	83.56	83.31	77.66	80.31
Variance, Mean, and Skewness	87.71	87.82	82.81	82.93	83.06	75.15	83.93
Variance, Mean, and Kurtosis	87.32	88.08	81.93	82.81	83.56	82.05	84.94
Kurtosis, Peak, and Slope	83.56	83.43	81.05	85.69	84.69	83.18	81.17
Kurtosis, Skewness, and Mean	43.78	50.43	56.33	53.19	48.68	52.94	48.05
Slope, Mean, and Skewness	49.68	48.68	48.93	53.95	55.58	51.69	49.32
Slope, Skewness, and Kurtosis	44.66	50.56	50.94	57.08	51.31	52.94	46.17
Slope, Mean, and Kurtosis	43.53	44.16	53.32	56.71	55.33	52.07	47.45
Slope, Skewness, and Variance	87.57	89.08	83.31	83.43	81.55	75.03	80.05
Skewness, Variance, and Kurtosis	87.95	88.83	82.55	83.18	82.81	84.19	86.57
Skewness, Peak, and Kurtosis	88.71	82.93	81.93	86.57	85.44	83.43	81.55
Skewness, Variance, and Peak	90.21	89.83	84.44	87.82	86.82	83.43	81.31

**Table 4:** **The classification accuracies of all 3-feature combinations obtained from HbR signals for all subjects**.

**Feature combination**	**S1**	**S2**	**S3**	**S4**	**S5**	**S6**	**S7**
Mean, Peak, and Slope	93.09	91.59	90.96	92.22	88.2	86.44	87.45
Mean, Peak, and Kurtosis	92.97	93.45	91.46	92.34	89.46	86.19	88.2
Mean, Peak, and Skewness	93.97	94.11	92.09	93.97	91.71	86.57	88.71
Mean, Peak, and Variance	93.47	92.84	90.33	92.09	88.83	88.08	92.09
Peak, Slope, and Skewness	88.95	89.08	84.56	86.07	85.94	83.68	84.18
Peak, Kurtosis, and Variance	87.07	88.08	84.06	88.45	87.07	78.16	84.56
Peak, Slope, and Variance	86.82	88.45	83.6	87.07	85.94	84.06	84.31
Variance, Slope, and Kurtosis	82.31	87.95	85.19	86.32	82.55	76.53	79.29
Variance, Slope, and Mean	79.17	87.45	82.05	85.82	82.81	79.42	79.67
Variance, Mean, and Skewness	82.31	86.82	83.18	86.57	83.18	80.05	78.79
Variance, Mean, and Kurtosis	86.07	87.07	85.19	86.44	83.43	79.92	79.54
Kurtosis, Peak, and Slope	87.57	86.44	81.55	86.32	85.82	83.43	84.94
Kurtosis, Skewness, and Mean	50.69	49.56	54.83	44.54	45.29	59.72	57.71
Slope, Mean, and Skewness	50.81	52.81	56.83	53.19	51.81	51.44	57.21
Slope, Skewness, and Kurtosis	48.18	56.83	52.82	47.81	45.54	56.71	59.84
Slope, Mean, and Kurtosis	49.18	55.45	52.19	45.04	49.05	61.11	58.09
Slope, Skewness, and Variance	80.42	86.71	83.34	86.71	81.31	76.41	77.66
Skewness, Variance, and Kurtosis	86.07	86.19	85.57	87.45	82.18	77.91	78.41
Skewness, Peak, and Kurtosis	89.48	85.14	86.07	84.23	85.08	84.24	85.44
Skewness, Variance, and Peak	88.33	89.58	87.45	89.83	87.95	78.67	82.05

## Discussion

**Figure 3:**
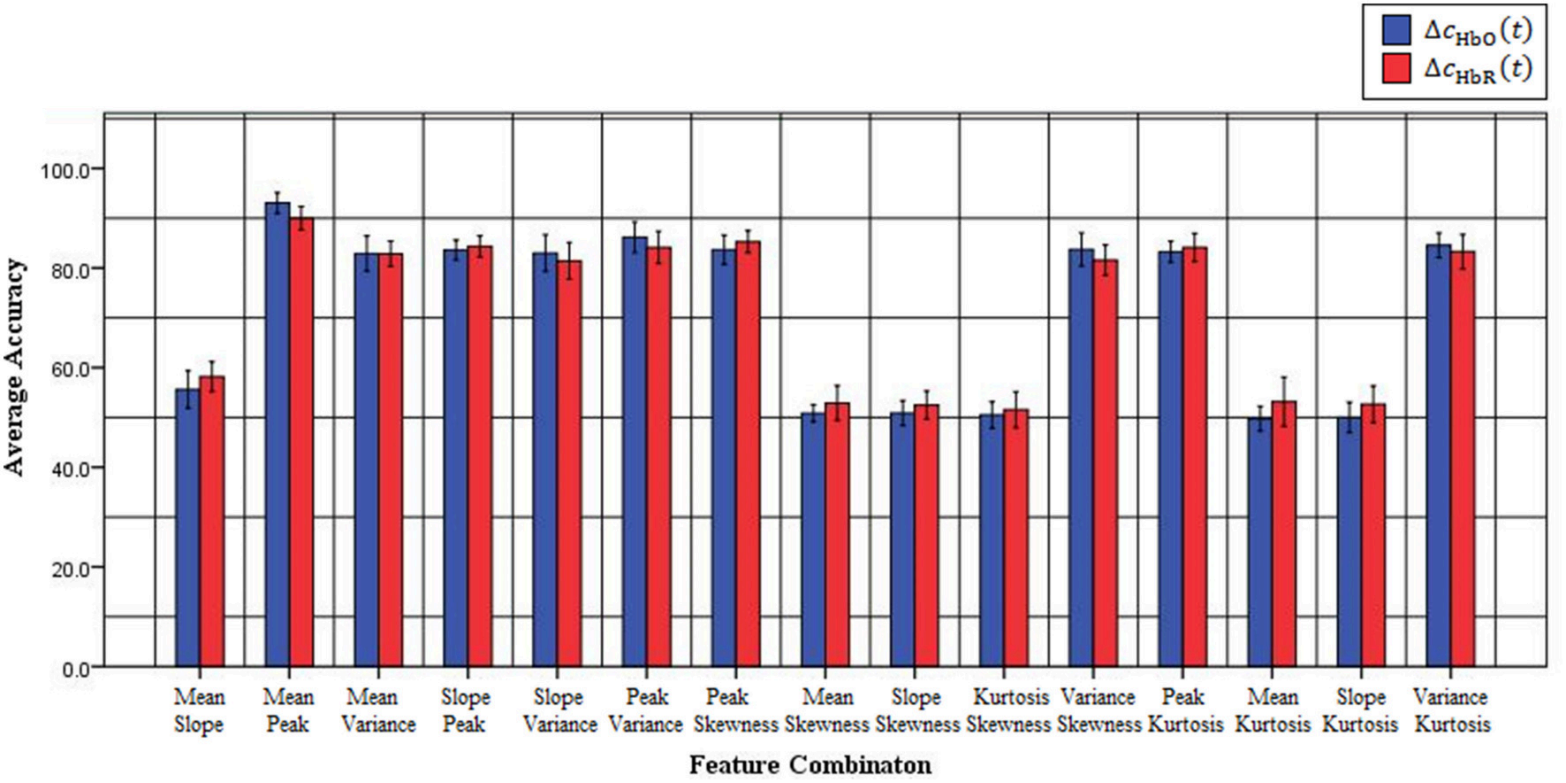
**Classification accuracies of all possible 2-feature combinations averaged across all subjects using △*c*_HbO_(*t*) and △*c*_HbR_(*t*)signals**.

**Figure 4:**
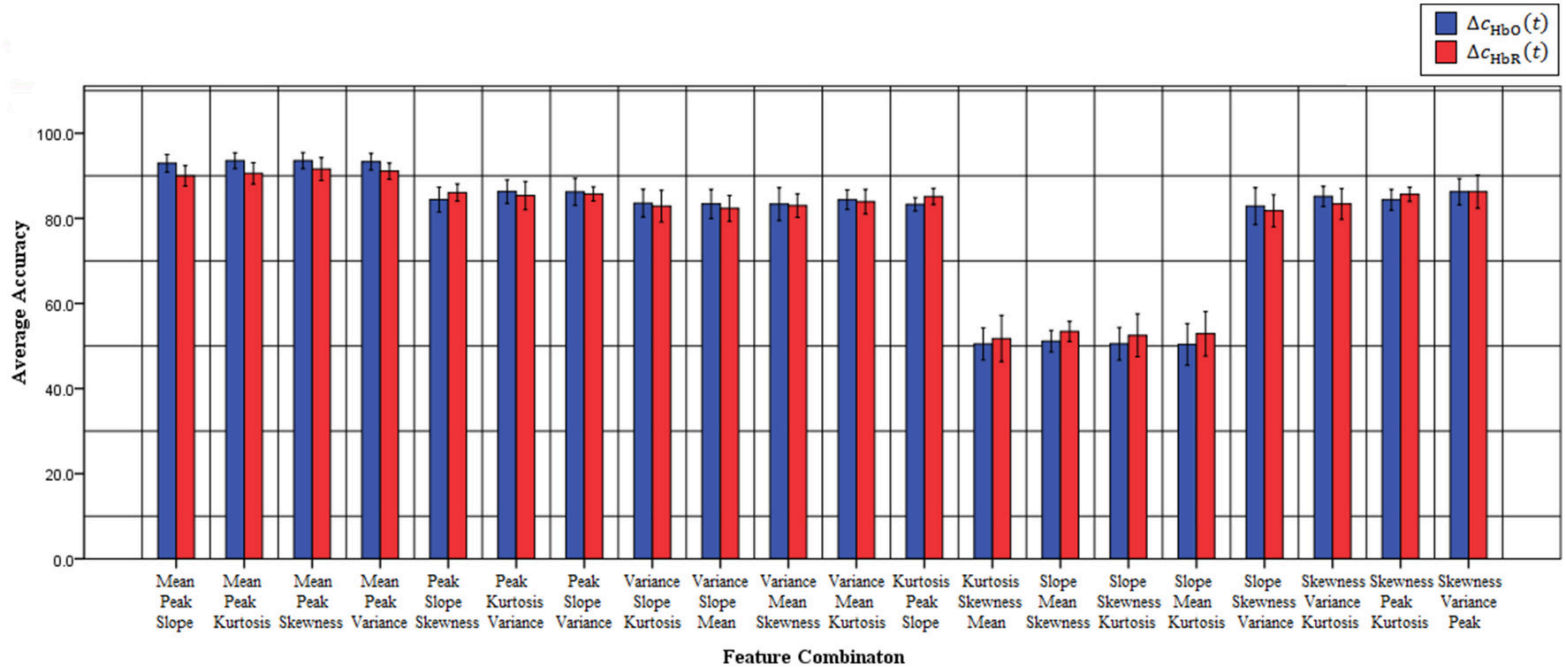
**Classification accuracies of all possible 3-feature combinations averaged across all subjects using △*c*_HbO_(*t*) and △*c*_HbR_(*t*) signals**.

Previous fNIRS-based BCI studies have mostly emphasized advanced signal-processing techniques and improved algorithms to improve classification accuracy and, thereby, enhance BCI performance (Sitaram et al., 2007; Power et al., 2010, 2011; Bauernfeind et al., 2011; Holper and Wolf, 2011; Abibullaev and An, 2012). These studies used mean (Sitaram et al., 2007; Power et al., 2010; Holper and Wolf, 2011; Faress and Chau, 2013; Naseer and Hong, 2013, 2015b; Power and Chau, 2013; Hong et al., 2014), variance (Tai and Chau, 2009; Holper and Wolf, 2011), slope (Tai and Chau, 2009; Power et al., 2011; Naseer and Hong, 2013, 2015b; Hong et al., 2014), kurtosis (Holper and Wolf, 2011), peak value (Tai and Chau, 2009; Cui et al., 2010a; Bauernfeind et al., 2011; Holper and Wolf, 2011) and skewness (Tai and Chau, 2009; Holper and Wolf, 2011) as features for classification (a detailed review of features used in the previous studies is provided in Naseer and Hong, 2015a). However, all of these feature might not contain discriminative information for classification and, therefore, in order to achieve high performance for fNIRS-based BCI systems different feature-combinations should be tested. In the current study, to improve the accuracy of “mental arithmetic vs. rest task” discrimination, various feature combinations were used to determine the single best combination for a two-class fNIRS-based BCI system. To the best of our knowledge, this is the first study to evaluate classification performance based on 2- and 3-feature combinations to determine those yielding the highest classification accuracies in discriminating mental arithmetic from rest tasks. The results demonstrate that the feature combination of peak and mean is the best among all possible combinations for both △*c*_HbO_(*t*) and △*c*_HbR_(*t*) signals. Furthermore, the peak-and-mean feature combination is the only one for which all subjects showed classification accuracies over 89%. Figure 3 plots the average classification accuracies of all *2-features combinations* across all subjects for △*c*_HbO_(*t*) and △*c*_HbR_(*t*) signals, respectively; Figure 4 plots the average classification accuracies of all *3-features combinations* across all subjects for △*c*_HbO_(*t*) and △*c*_HbR_(*t*) signals, respectively.

Mental arithmetic and rest have been classified using LDA in previous studies. For example, Naseer et al. (2014) classified mental arithmetic vs. rest with accuracies of 74.2% using mean values of △*c*_HbO_(*t*) and △*c*_HbR_(*t*). In the present study, the optimal combination yielded a higher, 93% accuracy. Khan and Hong (2015) used 2-feature combinations of 8 features (28 combinations of 7 HbO-based features and 1 HbR-based feature) to demonstrate the feasibility of using mean and peak values to achieve high classification accuracies of up to 84.6%. In our study, we used both 2- and 3-feature combinations of 6 HbO and 6 HbR features, thus making for a total of 70 (30 2-feature and 40 3-feature) combinations. With regard to the 3-feature combinations, those including mean and peak features yielded higher accuracies ranging from 92 to 94%. Furthermore, Khan and Hong's (2015) study was based on a passive driving-task BCI, whereas ours dealt with an active, arithmetic-task BCI.

This study has some limitations. The first is that only mean, peak, slope, variance, skewness and kurtosis, 2- and 3- feature combinations of HbO and HbR were used. The rationale, however, was that these features are the most commonly used in fNIRS-based BCI studies (Naseer and Hong, 2015a). The second limitation of our study is the small sample size and low number of subjects. Usually in fNIRS based BCI studies, 7–12 persons are considered enough for data acquisition (Penny et al., 2000; Hu et al., 2012; Zimmermann et al., 2013; Hong et al., 2014; Naseer and Hong, 2015b). However, using more subjects might be desirable to validate the findings. The third limitation is that only LDA is used to acquire classification accuracies and generalization to other classifiers is not done. However, the authors, in their future work, are working on finding the effects of using these optimal features on several other classifiers. The fourth limitation is that only two mental tasks (mental arithmetic and rest) were considered, which fact restricts the present study to a two-class BCI problem. For three-class BCI problems and above, other features and/or combinations might yield better results. Certainly, further research entailing multiple-mental-task classification using multiple-dimension optimal-feature combinations IS required.

## Conclusion

In this study we examined the effects of using different combinations of six commonly used features for classification of a two-class functional near-infrared spectroscopy (fNIRS)-based BCI based on mental arithmetic and rest tasks. It was shown that the combination of the peak and mean values of the changes in the concentrations of oxygenated hemoglobin (HbO) and deoxygenated hemoglobin (HbR) yielded the best average LDA-classification results for 2- as well as 3-feature sets across seven subjects. These results represent a step forward in the ongoing efforts to improve the classification accuracies of fNIRS-based BCI systems.

## Author contributions

NN conceived this study and was involved in experiments, data processing and writing of the manuscripts. FN and NQ performed the data analysis and wrote the manuscript. KH suggested the theoretical aspects of the current study and participated in revising the manuscript.

### Conflict of interest statement

The authors declare that the research was conducted in the absence of any commercial or financial relationships that could be construed as a potential conflict of interest.
